# Immunofluorescent staining reveals hypermethylation of microchromosomes in the central bearded dragon, *Pogona vitticeps*

**DOI:** 10.1186/s13039-015-0208-6

**Published:** 2015-12-30

**Authors:** Renae Domaschenz, Alexandra M. Livernois, Sudha Rao, Tariq Ezaz, Janine E. Deakin

**Affiliations:** Institute for Applied Ecology, University of Canberra, Canberra, ACT 2601 Australia; Present address: John Curtin School of Medical Research, The Australian National University, Canberra, ACT Australia; Discipline of Biomedical Sciences, Faculty of Education, Science, Technology and Mathematics, University of Canberra, Canberra, ACT 2601 Australia

**Keywords:** Reptiles, Methylation, Histone modifications, Epigenetics

## Abstract

**Background:**

Studies of model organisms have demonstrated that DNA cytosine methylation and histone modifications are key regulators of gene expression in biological processes. Comparatively little is known about the presence and distribution of epigenetic marks in non-model amniotes such as non-avian reptiles whose genomes are typically packaged into chromosomes of distinct size classes. Studies of chicken karyotypes have associated the gene-richness and high GC content of microchromosomes with a distinct epigenetic landscape. To determine whether this is likely to be a common feature of amniote microchromosomes, we have analysed the distribution of epigenetic marks using immunofluorescence on metaphase chromosomes of the central bearded dragon (*Pogona vitticeps*). This study is the first to study the distribution of epigenetic marks on non-avian reptile chromosomes.

**Results:**

We observed an enrichment of DNA cytosine methylation, active modifications H3K4me2 and H3K4me3, as well as the repressive mark H3K27me3 in telomeric regions on macro and microchromosomes. Microchromosomes were hypermethylated compared to macrochromosomes, as they are in chicken. However, differences between macro- and microchromosomes for histone modifications associated with actively transcribed or repressed DNA were either less distinct or not detectable.

**Conclusions:**

Hypermethylation of microchromosomes compared to macrochromosomes is a shared feature between *P. vitticeps* and avian species. The lack of the clear distinction between macro- and microchromosome staining patterns for active and repressive histone modifications makes it difficult to determine at this stage whether microchrosome hypermethylation is correlated with greater gene density as it is in aves, or associated with the greater GC content of *P. vitticeps* microchromosomes compared to macrochromosomes.

## Background

Epigenetic marks, such as DNA methylation and histone modifications, change the accessibility of DNA to the transcription machinery, thereby regulating gene expression. Most of our understanding of the role of epigenetic marks in vertebrates has been learnt from the study of model species such as mice, with far fewer studies having been carried out on non-model and non-mammalian species [1]. However, non-model species have genomic features that make them interesting to study from an epigenetic perspective [1]. For instance, the genome organisation of reptiles is quite different to that of mammals, with most species possessing several macrochromosomes and a varying number of microchromosomes [reviewed in 2]. This type of genome arrangement was most likely present in the ancestral amniote, and even in the tetrapod ancestor which diverged over 400 million years ago [3]. The conservation of this division between macro- and microchromosomes over a long evolutionary timescale makes it interesting to characterize the similarities and differences between the two types of chromosomes, including the distribution of epigenetic marks.

Our general understanding of microchromosomes in vertebrates is rather limited considering the number of species in which they are found. Cross-species chromosome painting and gene mapping amongst avian species demonstrate, in most cases, that a microchromosome in one species is conserved as a microchromosome in another [4–7], indicating that microchromosomes are fairly conserved amongst aves. Whole genome sequencing has enabled detailed sequence analysis of chicken microchromosomes and comparisons of genomic features between macro- and microchromosomes. Chicken microchromosomes are early replicating [8], higher in gene density [9, 10], GC and CpG content [11, 12], recombination rate [9] and rate of synonymous substitutions [13] but are lower in repeat content than macrochromosomes [9]. In keeping with the higher CpG content, DNA methylation is enriched on microchromosomes of chicken, quail, pheasant, emu and American rhea [4]. Histone modifications H4K5ac and H4K8ac, associated with actively transcribed DNA, are also enriched on chicken microchromosomes and thought to correlate with the high gene density [8, 14].

Although genes from some chicken microchromosomes are located on macrochromosomes in reptiles [15–17], the smaller number of microchromosomes present in non-avian reptiles display conserved synteny with avian microchromosomes [17]. This has been demonstrated by whole genome sequencing of the green anole lizard genome [17] and comparative gene mapping in other species [3, 15, 16, 18, 19], dating these microchromosomes back to at least the amniote ancestor [3]. However, it appears that the characteristics of chicken microchromosomes may not be conserved across all reptiles. For instance, there is no difference in GC content between anole lizard macrochromosomes and six of the 12 pairs of microchromosomes for which sequence has been assigned [17], although the central bearded dragon [20, 21], tuatara [22] Japanese four-striped rat snake [18] and soft shelled turtle microchromosomes are more GC rich than macrochromosomes [23]. This raises questions whether the epigenetic differences observed between macro- and microchromosomes in chicken would also be observed in non-avian reptiles.

The central bearded dragon (*Pogona vitticeps*) is an Australian lizard species for which there are considerable genetic and genomic resources available, including a molecular cytogenetic map [24] and genome sequence [21]. This species has a diploid chromosome number of 32, consisting of 6 pairs of macrochromosomes and 10 pairs of microchromosomes [25]. A pair of microchromosomes were discovered to be the sex chromosomes in this species, possessing a ZZ male:ZW female sex chromosome system with a highly heterochromatic W chromosome [20].

Here we report the occurrence of DNA methylation as well as two active and two repressive histone modifications on *P.vitticeps* metaphase chromosomes using immunofluorescent staining. This approach is particularly valuable for non-model species where genome sequences lack adequate sequence coverage for a high quality genome assembly to be used as a reference genome for sequence-based approaches like ChIP-seq or bisulfite sequencing. In addition, although these sequencing-based techniques provide valuable, fine-scale information, these data typically represent the mean occurrence of an epigenetic mark from heterogeneous cells, with possible differences between cells arising from them being at different stages of the cell cycle [26]. Immunofluorescent staining of epigenetic modifications on metaphase chromosomes allows the distribution of epigenetic marks along individual chromosomes, including the difficult to sequence repetitive regions, to be examined within a single cell.

The active histone modifications we have chosen are histone H3 di-methylated at lysine 4 (H3K4me2) and H3 tri-methylated at lysine 4 (H3K4me3), which are epigenetic marks typically associated with euchromatin and are closely associated with gene-rich regions of the genome, CpG islands and SINE elements on human chromosomes [26]. In contrast, histone H3 tri-methylated at lysine 27 (H3K27me3) is a repressive epigenetic mark associated with facultative heterochromatin and the repression of gene transcription. The other repressive mark we used is histone H3 di-methylated at lysine 9 (H3K9me2) which is associated with constitutive heterochromatin formation as well as being involved in gene regulation during development (reviewed in [27]).

## Results and discussion

We compared the distribution of epigenetic marks between macro- and microchromosomes, using immunofluorescent staining to determine if there is an epigenetic distinction between the two different categories of chromosomes. Despite this technique being a valuable tool to study the epigenetic state of chromosomes for non-model species, there have been very few studies that have employed this approach for non-model vertebrates. Using this approach, we detected obvious staining differences between macro- and microchromosomes for 5-methylcytosine staining but not for active or repressive histone modifications.

### DNA methylation status

Immunostaining with a 5-methylcytosine (meC) antibody was used to visualize the global DNA methylation state of metaphase chromosomes. Telomeric regions of most *P.vitticeps* chromosomes showed stronger methylation staining than the rest of the chromosome, a pattern that has been observed in a range of species such as human [28], Tasmanian devil [29], platypus [30] and even plants [31]. The telomeric repeat sequence (TTAGGG)n in vertebrates does not contain the CG dinucleotide required for methylation to occur. However, adjacent subtelomeric regions in mammals are GC rich and heavily methylated [32], with methylation of these regions implicated in repressing DNA recombination at telomeres and indirectly regulating telomere length [33]. With the important role telomeres play in protecting the ends of chromosomes from eroding, it is not surprising that methylation of telomeric/subtelomeric regions may not be restricted to mammals.

**Fig. 1:**
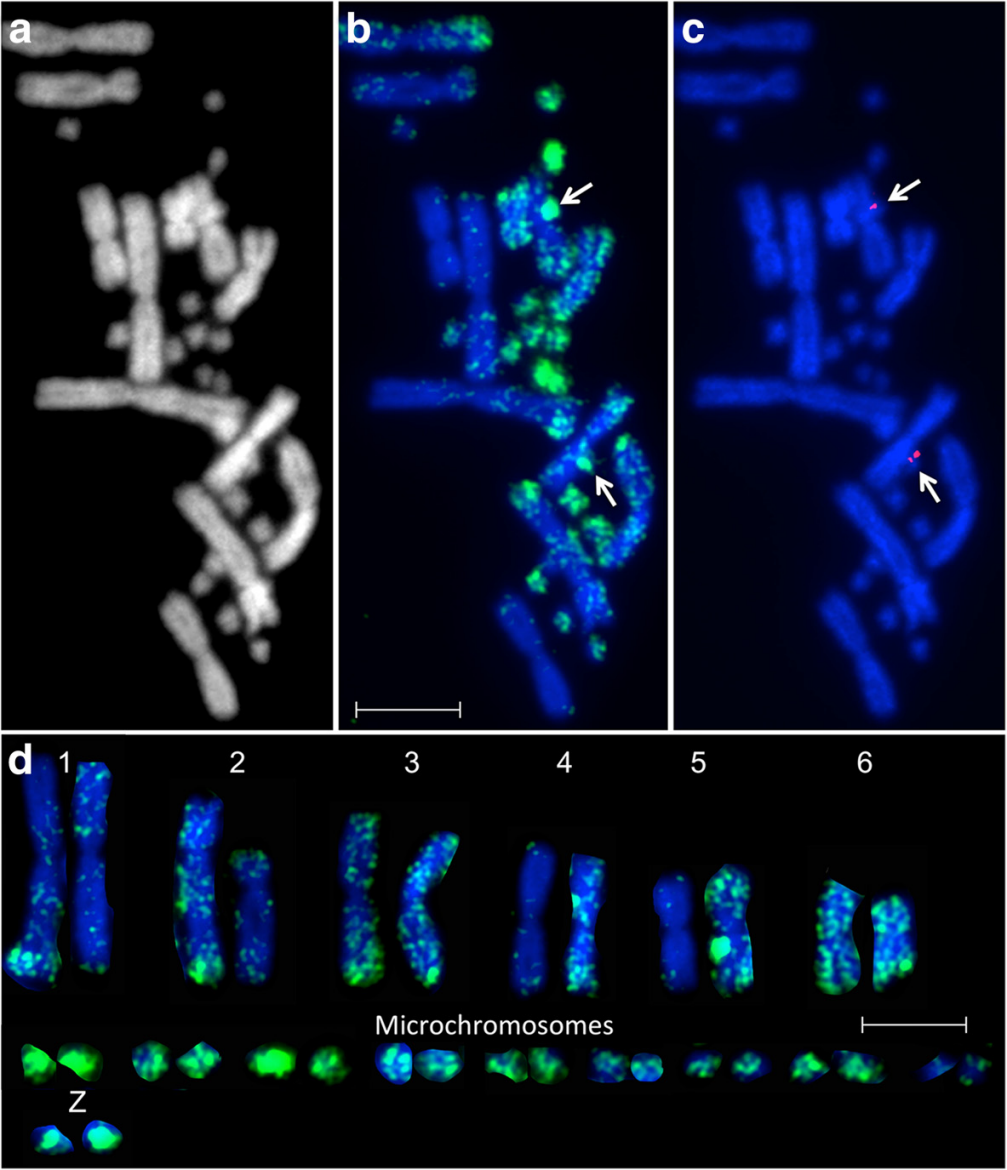
Methylation patterns on male *Pogona vitticeps* metaphase chromosomes. Images for (**a**) DAPI, (**b**) DNA methylation (5-meC), and (**c**) identification of the Z chromosomes by mapping of BAC 150H19 specific to the sex chromosomes. **d** Karyotype of chromosomes depicted in images a-c. Scalebars represent 10 μm

All observed metaphase spreads from both cell lines examined showed a more intense staining of microchromosomes than macrochromosomes (Fig. 1). This is consistent with the observation that *P. vitticeps* microchromosomes are GC rich [20], as well as the methylation pattern observed in avian species, suggesting that, like birds, *P. vitticeps* microchromosomes are gene rich. Grützner et al. [4] proposed that, given the known role of methylation in gene silencing, higher levels of methylation on the gene-dense avian microchromosomes may indicate that most genes are inactive in any given cell. This may be true if DNA methylation was solely associated with gene silencing, but hypermethylation of gene bodies is associated with gene activity [34, 35]. Thus, hypermethylation of microchromosomes may be correlated with gene activity of these gene rich chromosomes. Alternatively, the seemingly more intense staining of microchromosomes may simply be attributed to the closeness of the telomeric regions on these tiny chromosomes and DNA methylation may not be an indicator of their gene activity. A sequencing-based approach could prove useful for distinguishing between these alternative explanations for hypermethylation of *P. vitticeps* microchromosomes.

In mammals, inactivation of one X chromosome in females compensates for the differences in dosage of X-borne genes between XX females and XY males. In marsupials and humans, the inactive X in females is hypomethylated compared to the active X and autosomes, most likely as a result of gene-body methylation which is associated with gene activity [30, 35, 36]. As the sex chromosomes in *P. vitticeps* are microchromosomes, we carefully examined male metaphase spreads for a hypomethylated Z chromosome. However, both copies of the Z chromosome were consistently hypermethylated in males (Fig. 1). This suggests, that if there is a mechanism in *P. vitticeps* to compensate for the difference in Z gene dosage between ZZ males and ZW females, it is unlikely to be similar to the chromosome-wide mechanism observed in therian (marsupial and eutherians) mammals.

### Active modifications

**Fig. 2:**
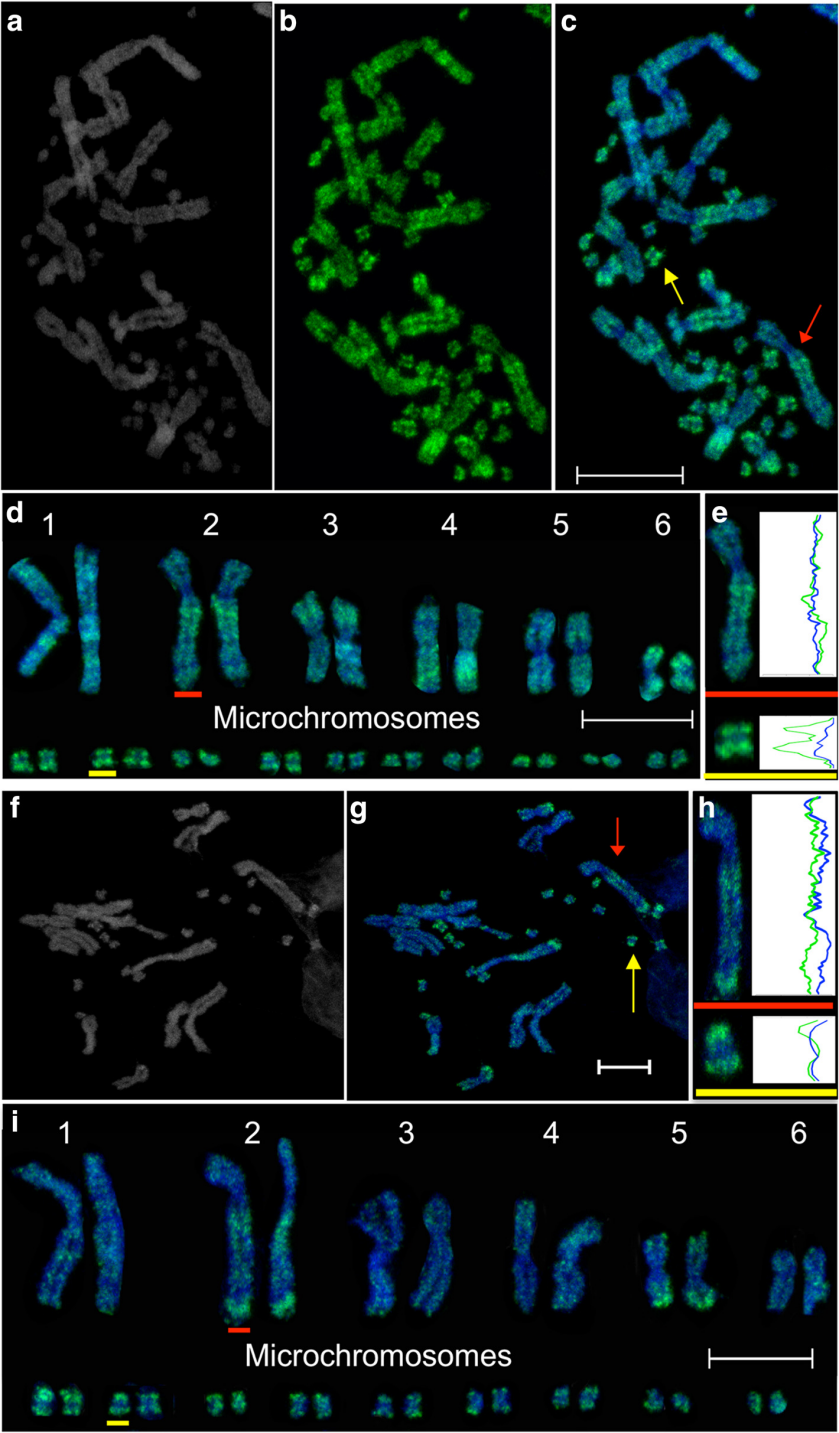
Immunofluorescent staining of active marks H3K4me2 and H3K4me3 on *Pogona vitticeps* metaphase chromosomes. Distribution of H3K4me2: (**a**) DAPI stained chromosomes, (**b**) H3K4me2 staining and (**c**) merged image, (**d**) karyotype of chromosomes depicted in image c. **e** Representative line scans of staining on a macrochromosome (red) and microchromosome (yellow). The blue curves correspond to the DAPI staining along the length of the chromosomes. The green curves show the distribution of each epigenetic mark. Distribution of H3K4me3: (**f **) DAPI stained chromosomes, (**g**) merged image showing H3K4me3 staining in green and DAPI staining in blue. **h** Representative line scans of staining on a macrochromosome (red) and microchromosome (yellow). **i** Karyotype of chromosomes depicted in image g. Scalebars represent 10 μm

**Fig. 3:**
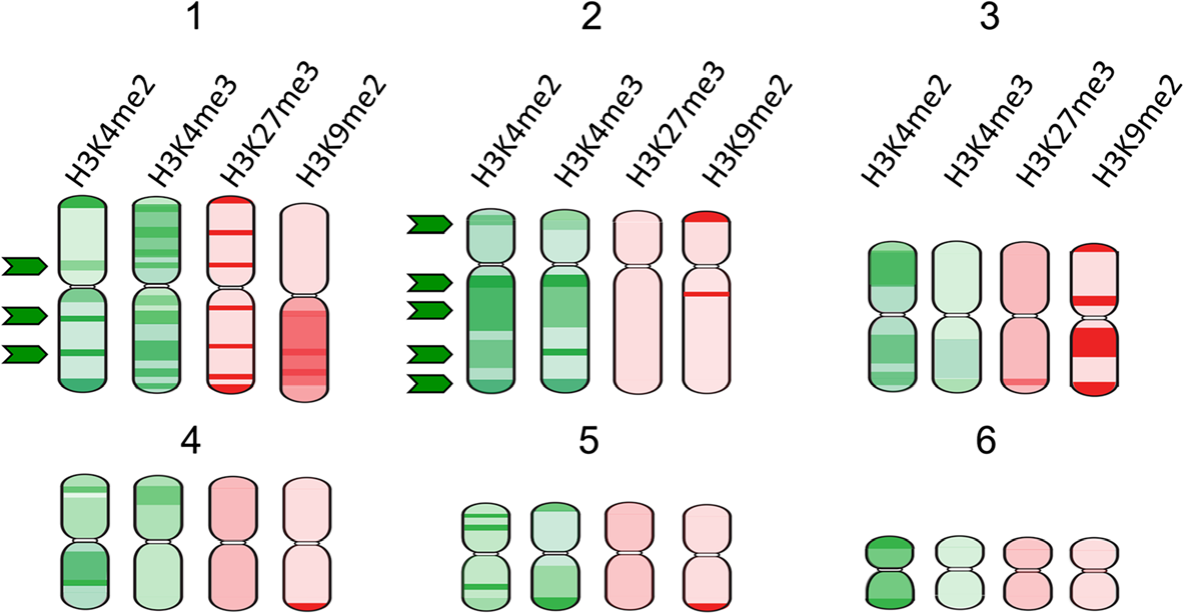
Ideograms depicting the distribution of active marks (green) H3K4me2 and H3K4me3 and repressive marks (red) H3K27me3 and H3K9me2 on *Pogona vitticeps* macrochromosomes. Arrows indicate regions of overlap between the two active marks

In *P. vitticeps*, distribution of H3K4me2 (Fig. 2a-e) and H3K4me3 (Fig. 2f-i) staining across telomeric regions and on both arms of macrochromosomes was seen with a distinct pattern for each macrochromosome. Although the distributions of these two active marks are different, there is some overlap of intensely stained regions on chromosomes 1 and 2 (Fig. 3). These regions are likely to represent particularly gene-rich regions of the genome. In humans, H3K4me3 enriched regions on chromosomes have been shown to correspond with gene-rich regions [26].

Although the fragile nature of the unfixed chromosomes [37] from primary fibroblast cell lines made karyotyping of all microchromosomes in a metaphase spread challenging, the majority of microchromosomes were consistently detected to gain a general impression of the distribution of these active marks. The line scans (Fig. 2e and h) demonstrate enrichment for H3K4me2 and H3K4me3 in telomeric regions and an absence from the centromeric/pericentric regions of microchromosomes. Like the pattern observed for DNA methylation, it is unclear whether the enrichment for these marks is correlated with gene activity on potentially gene rich microchromosomes or due to the proximity of the telomeres on the tiny chromosomes. Telomeric enrichment of these marks, also observed for human telomeres, may be associated with RNA polymerase II transcription reported at mammalian telomeres [38–40]. In contrast, centromeric chromatin on *P.vitticeps* chromosomes was consistently unstained for H3K4me2 and H3K4me3, which is expected given the heterochromatic nature of centromeres.

### Repressive modifications

**Fig. 4:**
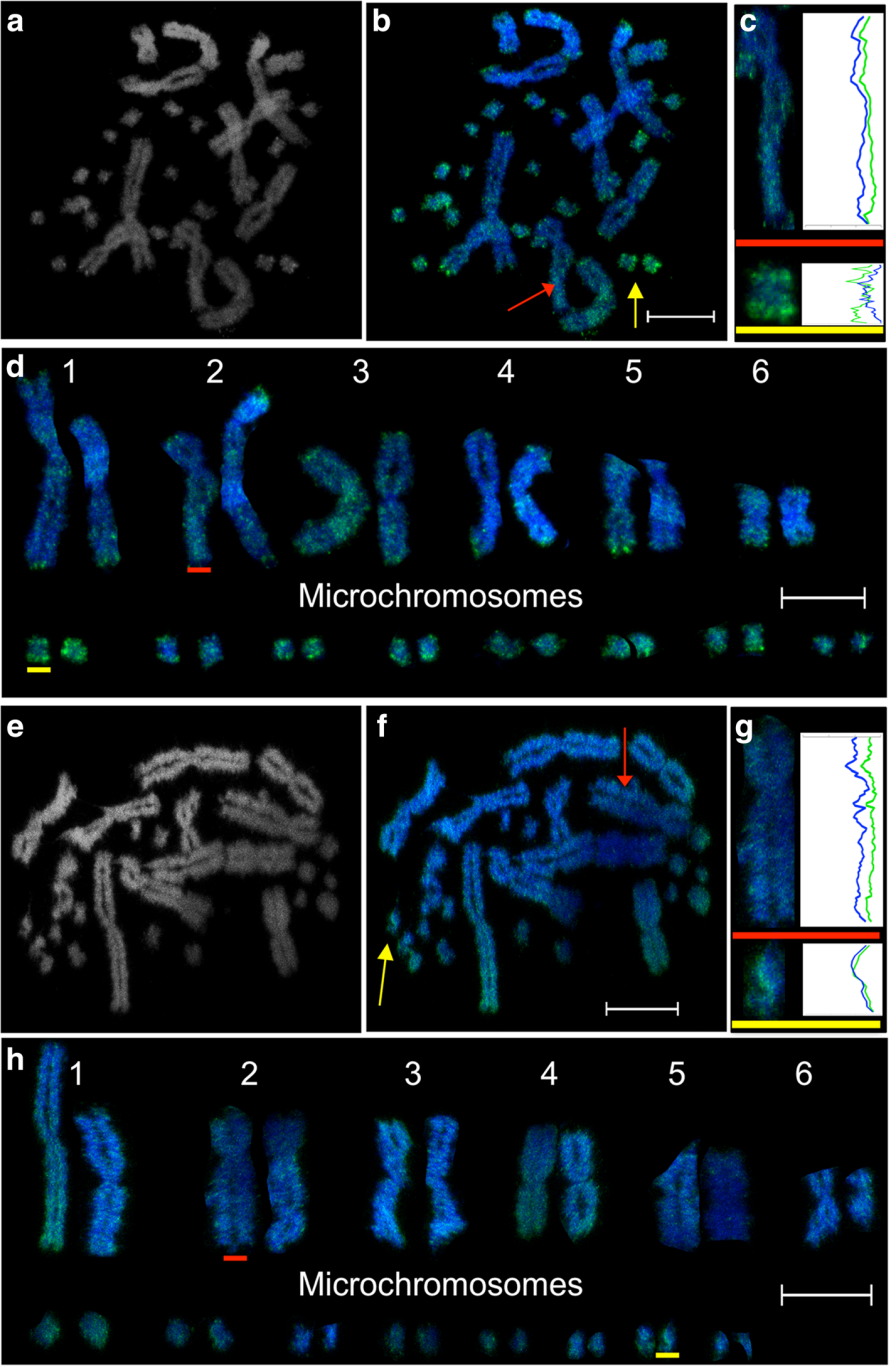
Distribution of repressive epigenetic marks across *Pogona vitticeps* metaphase chromosomes. Distribution of H3K27me3: (**a**) DAPI stained chromosomes, (**b**) merged image with H3K27me3 staining in green and DAPI in blue, (**c**) Representative line scans of staining on a macrochromosome (red) and microchromosome (yellow). The blue curves correspond to the DAPI staining along the length of the chromosomes. The green curves show the distribution of H3K27me3. **d** Karyotype of chromosomes depicted in image b. Distribution of H3K4me3: (**e**) DAPI stained chromosomes, (**f**) merged image showing H3K9me2 staining in green and DAPI staining in blue. **g ** Representative line scans of staining on a macrochromosome (red) and microchromosome (yellow). **h** Karyotype of chromosomes depicted in image g. Scalebars represent 10 μm

The modification H3K27me3, associated with gene silencing, showed a distinctive regional distribution along the arms of macrochromosomes, with intense staining detected in 70–80 % of the metaphase spreads in telomeric regions (Fig. 4a-d). H3K27me3 was also strongly enriched at telomeric regions of microchromosomes, a staining pattern we also observed with active modifications H3K4me2 and H3K4me3 as earlier described. Enrichment for H3K27me3 staining at telomeric regions has also been observed on human metaphase chromosomes [26]. Like macrochromosomes, centromeric chromatin was unstained for H3K27me3 on microchromosomes. Also associated with gene silencing, H3K9me2 showed an evenly distributed and much less defined staining pattern along the arms of both macro- and microchromosomes than H3K27me3. (Figure 4e-h). Antibodies for these two histone modifications have shown a similar pattern of staining on tammar wallaby (*Macropus eugenii*) metaphase chromosomes [41]. There is a lack of overlap of regions enriched for these two repressive marks (Fig. 3), which is not surprising given that one is associated with facultative heterochromatin (H3K27me3) and the other with constitutive heterochromatin.

## Conclusions

We show a characteristic distribution of various histone modifications across the metaphase genome of *P. vitticeps*, with some modifications showing distinctive regional localisation. DNA cytosine methylation, active modifications H3K4me2 and H3K4me3, as well as the repressive mark H3K27me3 are enriched in telomeric regions. The most notable epigenetic difference between macro- and microchromosomes is the hypermethylation of microchromosomes, a feature shared with birds. None of the histone modifications examined showed as distinct a difference between macro- and microchromosomes as DNA cytosine methylation. The lack of difference between macro- and microchromosomes for histone modifications associated with gene activity makes it unclear whether this difference is correlated with increased gene density, as it is in avian species, or simply a reflection of the increased GC content or closeness of the methylation staining associated with telomeric regions of *P. vitticeps* microchromosomes. With the sequencing of more reptile genomes, including that of *P. vitticeps*, it will be interesting to compare the genomic features of macro- and microchromsomes to their epigenetic signature.

## Methods

### Cell culture

Primary adult *P.vittceps* fibroblast cell lines were derived as previously described [42] from samples collected under approval from the University of Canberra Committee for Ethics in Animal Experimentation (CE-04-04). Cultured cells were maintained in Gibco AmnioMax medium (Life Technologies Australia Pty Ltd, Mulgrave, VIC, Australia), supplemented with 10 % fetal bovine serum (Autogene Bioclear, Calne, Wiltshire, UK), 1 mM L-glutamine (Gibco-BRL, Life Technologies), 50 U/ml penicillin (Gibco-BRL, Life Technolgies), and 50 μg/ml of streptomycin (Gibco-BRL, Life Technologies). Cells were grown at 28 °C in an atmosphere containing 5 % CO_2_.

### Immunostaining for DNA methylation

Metaphase slides were prepared using standard protocols [43]. The slides were dehydrated through 70 %, − 90 % - 100 % (v/v) ethanol series (3 min each) and air dried before denaturing in 70 % (v/v) formamide at 70 °C for 1 min and 40 s. The slides were immediately transferred to ice-cold 70 % (v/v) ethanol for 5 min and then continued through 90 and 100 % (v/v) ethanol series (3 min in each). The slides were allowed to air dry before rehydrating in Phosphate Buffered Saline with Tween 20 (PBST: 137 mM NaCl, 2.7 mM KCl, 10 mM NA_2_HPO_4_, 2 mM 2.4 KH_2_PO_4_, 0.03 % v/v Polysorbate 20) for 3 min. The slides were blocked in PBST + 1 %(w/v) Bovine Serum Albumin (BSA) for 20 min, after which the primary anti-5-methylcytosine antibody (5meC), diluted 1:200 in PBST, was added to the slides and incubated for 60 min in a humidified chamber at 37 °C. Subsequently, the slides were washed twice for 5 min each in PBST. The area was then covered with the secondary antibody (anti-mouse Cy3) diluted 1:500 in PBST, and incubated for 60 min in a humidified chamber at 37 °C. The slides were then fixed in 4 % (w/v) paraformaldehyde in PBS for 15 min, washed in PBST 3 times for 3 min each, air dried and mounted in Vectashield with 4′-6-diamidino-2-phenylindole (DAPI) (Vector Laboratories Inc., Burlingame, CA, USA). Fluorescent staining was visualized using a Zeiss Axio Scope A1 epifluorescence microscope and captured on an AxioCam Mrm Rev.3 CCD (charge-coupled device) camera (Carl Zeiss Ltd) using Isis FISH Imaging System version 5.4.11 software (MetaSystems, Newton, MA, USA). At least ten metaphase spreads were captured for each cell line. Line scans of DAPI and methylation staining intensities were obtained using Image-Pro Plus software (MediaCybernectics).

### Fluorescent in situ hydridisation (FISH)

To identify the sex chromosomes, fluorescent in situ hybridization (FISH) was performed on the same slides as the 5meC staining using a BAC clone known to map to the sex chromosomes. The slide was prepared for FISH by rinsing in 2 × saline sodium citrate (SSC) buffer (0.3 M NaCl, 0.03 M sodium citrate, pH7) and dehydrating it through a 70 % (v/v), 90 % (v/v), 100 % (v/v) ethanol series. DNA for BAC clone Pv_150H19 known to map to the sex chromosomes was extracted using the WIZARD SV Minipreps DNA Purification System (Promega, Alexandria, NSW, Australia). The DNA was fluorescently labelled by nick translation with SpectrumOrange dUTP (Abbott Molecular Inc., Des Plaines, IL, USA) and hybridised as previously described [43]. Unbound probe was removed as described by Deakin et al. [44] and fluorescent signals visualised and captured using the same microscope, camera and software as that used for the detection of 5meC staining.

### Immunofluorescence detection of histone modifications

Colcemid (Roche, Castle Hill, Australia) was added to the cell cultures at a final concentration of 0.1 μg/ml before harvesting metaphase chromosomes. Cells were harvested by trypsinization, collected in culture medium, and hypotonized in 0.0375 M KCl for 10 min at room temperature. Samples (0.15 ml) of the hypotonic cell suspension were cytospun onto clean glass slides in the presence of 10 % Tween 20 (3ul) at 800–1,200 rpm for 6 min. The slides were treated with KCM buffer (120 mM KCl, 20 mM NaCl, 10 mM Tris/HCl pH 8.0, 0.5 mM EDTA, 0.1 % Triton X-100) plus 1 % bovine serum albumin for 5 min at room temperature and rinsed in KCM buffer twice before immunostaining. The slides were incubated in a humidified chamber at room temperature with primary antibodies for 2 h, and secondary antibodies for 1 h. Primary and secondary antibodies are listed in Table 1. Each incubation with antibodies was accompanied by washing in KCM buffer (3 × 5 min). After the last washing, the slides were counterstained with DAPI, fixed in 4 % paraformaldehyde (w/v) for 10 min at room temperature, and mounted in Vectashield mounting medium (Vector Laboratories). The chromosomes were visualized using a Nikon Eclipse Ti fluorescence microscope and NIS Elements AR software. For each chromatin modification, at least 10 metaphases of the primary culture were analyzed. The line scans of DAPI and histone modification intensities were obtained using Image-Pro Plus software (MediaCybernectics).

**Table 1:** Primary and secondary antibodies used for immunofluorescence

Antibodies	Raised/type	Source	Catalog no.
Anti-5-methylcytosine (5meC) (Clone 10G4)	Mouse monoclonal	Zymo	A3001
Anti-H3K4me2	Rabbit polyclonal	Upstate (Millipore)	07–030
Anti-H3K27me3	Rabbit polyclonal	Upstate (Millipore)	07–449
Anti-H3K9me2	Rabbit polyclonal	Upstate (Millipore)	07–441
Anti-H3K4me3	Mouse monoclonal	Abcam	ab–1012
Anti-Cy3 anti-mouse	Donkey polyclonal	Jackson Immunoresearch Laboratories	715–165–151
Anti-FITC anti-rabbit	Donkey polyclonal		711–095–152

## Abbreviations

CCD: Charge-coupled device
DAPI: 4′–6-diamidino-2-phenylindole
FISH: Fluorescent in situ hybridization
H3K4me2: Histone H3 di-methylated at lysine 4
H3K4me3: H3 tri-methylated at lysine 4
H3K27me3: Histone H3 tri-methylation at lysine 27
H3K9me2: Histone H3 di-methylation of lysine 9
meC: 5-methylcytosine
PBST: Phosphate buffered saline with Tween 20
